# Associating transcriptional regulation for rapid germination of rapeseed (*Brassica napus* L.) under low temperature stress through weighted gene co-expression network analysis

**DOI:** 10.1038/s41598-018-37099-0

**Published:** 2019-01-11

**Authors:** Tao Luo, Mengzhu Xian, Chen Zhang, Chunni Zhang, Liyong Hu, Zhenghua Xu

**Affiliations:** MOA Key Laboratory of Crop Ecophysiology and Farming System in the Middle Reaches of the Yangtze River/College of Plant Science and Technology, Huazhong Agricultural University, Wuhan, 430070 China

## Abstract

Slow germination speed caused by low temperature stress intensifies the risk posed by adverse environmental factors, contributing to low germination rate and reduced production of rapeseed. The purpose of this study was to understand the transcriptional regulation mechanism for rapid germination of rapeseed. The results showed that seed components and size do not determine the seed germination speed. Different temporal transcriptomic profiles were generated under normal and low temperature conditions in genotypes with fast and slow germination speeds. Using weight gene co-expression network analysis, 37 823 genes were clustered into 15 modules with different expression patterns. There were 10 233 and 9111 differentially expressed genes found to follow persistent tendency of up- and down-regulation, respectively, which provided the conditions necessary for germination. Hub genes in the continuous up-regulation module were associated with phytohormone regulation, signal transduction, the pentose phosphate pathway, and lipolytic metabolism. Hub genes in the continuous down-regulation module were involved in ubiquitin-mediated proteolysis. Through pairwise comparisons, 1551 specific upregulated DEGs were identified for the fast germination speed genotype under low temperature stress. These DEGs were mainly enriched in RNA synthesis and degradation metabolisms, signal transduction, and defense systems. Transcription factors, including WRKY, bZIP, EFR, MYB, B3, DREB, NAC, and ERF, are associated with low temperature stress in the fast germination genotype. The aquaporin NIP5 and late embryogenesis abundant (LEA) protein genes contributed to the water uptake and transport under low temperature stress during seed germination. The ethylene/H_2_O_2_-mediated signal pathway plays an important role in cell wall loosening and embryo extension during germination. The ROS-scavenging system, including catalase, aldehyde dehydrogenase, and glutathione S-transferase, was also upregulated to alleviate ROS toxicity in the fast germinating genotype under low temperature stress. These findings should be useful for molecular assisted screening and breeding of fast germination speed genotypes for rapeseed.

## Introduction

Seed germination is a crucial developmental stage in the life cycle of plants, beginning with the uptake of water by the quiescent dry seeds and ending with the protrusion of radicle through the seed coat^1,2^. Seed germination is accompanied by sequential and dynamic gene expression, protein synthesis, and post-translational modifications, which determine its potential for rapid and uniform emergence and seedlings establishment^3^. Germination speed, defined as the time taken for a non-dormant seed to germinate, is an important trait that determines the competitiveness and fitness of the plants in various environments^4^. Germination speed is sensitive to the surrounding environment, and it has been widely reported that germination is delayed by abiotic stresses, including drought^5–7^, low temperature^8–10^, salinity stress^5,11^, and heavy metal toxicity^12^.

The rice–rapeseed multiple cropping system is a long-established major crop production system in the Yangtze River basin^13^. The traditional method of seedling transplantation, which demands high labor and cost, has now been replaced with a direct-seeding method for rapeseed production in this area^14^. Under this new cultivation method, and particularly for late-sown croplands, low temperature can severely impede seed germination and limit the geographical distribution of rapeseed. Germination speed decreases at temperatures below the optimal germination range (20–25 °C) for rapeseed (*Brassica napus* L.)^15^. This delay of germination intensifies the adverse effects of other abiotic factors on germination, thus, lowering the final seedling emergence rate and limiting the ability of plants to produce a high seed yield^16^. The germination speeds under low temperature stress vary significantly among rapeseed genotypes (*Brassica napus* L.)^16–18^. The broad-sense heritability estimating for rapid germination at low temperatures are approximately 60% for rapeseed (*Brassica napus* L), and this trait follows a complex (possibly polygenic) inheritance pattern^19^. Selection for fast germination genotypes at low temperatures might improve germination characteristics over a range of temperatures^17,20^.

Previous studies have uncovered valuable information on the germination process under low temperature stress. Low temperature slows down enzyme kinetics, and consequently, cellular processes. Germination is an energy-demanding process, which requires functioning mitochondria that can remain active and stable through the assembly of proteins into membranes during imbibition^21^. Exposure to low temperature might decrease the respiratory activity of the cotyledons. For rapeseed, improved germination rates under low temperatures have been associated with high isocitrate lyase activities and rapid mobilization of total lipid and protein reserves^22,23^. With the application of next-generation sequencing (NGS) technology, several regulatory networks and related candidate genes for low-temperature-response have been elucidated, including hormonal responses to abscisic acid (ABA) and gibberellins (GA)^24–27^, ROS signal transduction pathway^28^, and APETALA2/Ethylene Responsive Factor (AP2/ERF) transcription factors^29,30^. Candidate genes controlling seed germination and vigor in *Brassica napus* L. have been identified by genome-wide association mapping^31^; however, studies on gene regulatory networks related to fast germination under low temperature stress remain rare^32–34^.

Therefore, there is a need for a comprehensive and systematic study of the response of the germination mechanism to low temperature. Correlation of gene expression is a powerful approach to analyze large datasets, and co-expressed genes tend to have a high topological overlap as they have an increased likelihood of being involved in the same biochemical/developmental pathways^35,36^. Utilizing the weighted gene co-expression network analysis^37,38^, the present study highlights the power of transcriptome analysis to elucidate specific network regulation of germination processes and to detect potential candidate genes involved in fast germination of rapeseed (*Brassica napus* L.) under low temperature stress.

## Material and Methods

### Experimental materials

The study tested 109 varieties (*Brassica napus* L.) originating from the Yangtze River basin in China. For this experiment, newly harvested seeds of each variety were collected from plant community and stored in a seed-storage cabinet, maintained at 23 °C and 8% humidity. Their seed quality indexes including linoleic acid (%), linolenic acid (%), oil content (%), oleic acid (%), palmitic acid (%), paullinic acid (%), protein content (%), stearic acid (%) and thioglycoside (μmol/g) were analyzed by using a Near Infrared Reflectance Spectroscopy System (NIRSystem 3750, Sweden) with three replicates of intact seeds weighted about 8 g. The thousand seed weights were also measured with three replicates for these varieties.

### Germination trials

The germination trials were conducted under low-temperature and normal-temperature conditions for these varieties. For each variety, intact and uniform seeds were selected and soaked in 0.1% sodium hypochlorite for 15 min, then washed four times in distilled water. After this treatment, the seeds were allowed to dry at room temperature. For each variety, three germination boxes were placed in a temperature-controlled incubator (day/night temperature cycle of 25/20 °C) with a 12-h photoperiod (150 μmol photons m^−2^ s^−1^), and another three germination boxes were placed in another temperature-controlled incubator (day/night temperature cycle of 15/10 °C) with the same light conditions. The temperature fluctuations in incubator were controlled within ± 1 °C. One-hundred seeds (arranged as 10 × 10) were sown in each germination box (12 cm × 12 cm × 6 cm) and covered with three layers of sterilized filter paper. The filter papers in each box were saturated with 10 ml distilled water, and 1 ml distilled water was added each day during the experimental period to provide adequate water for seed germination. The number of germinated seeds in each germination box were counted once daily for 2 weeks. The mean germination time (MGT) was calculated using the formula described by Matthews and Hosseini^39^ for each variety under normal and now temperature conditions, and larger MGT value corresponds to the slower germination speed. The correlation analysis between the MGT and seed quality of rapeseed varieties were performed using R package psych 1.5.8.

### Germination treatment and sampling of selected genotypes for transcriptome

A fast germinating genotype Ganyouza No. 5 and a slow germinating genotype Huawanyou No. 4 were screened out to generate transcriptomic profiles based on their performance at germination trials (Additional file 1). The two selected hybrid varieties (Ganyouza No. 5 and Huawanyou No. 4) were provided by the Jiangxi Academy of Agricultural Sciences Institute of Crops and Huazhong Agricultural University, respectively; the seeds were produced in the middle region of Yangtze River basin. The treatment conditions and management were same as the germination trials. For each variety, eighteen germination boxes were separately placed in normal- and low-temperature controlled incubators. Based on the germination speed of these two varieties, the fast-germination variety (FAG) was sampled at 1, 2, and 3 days after imbibition (DAI) under normal and low temperature treatment conditions. At each time point, three boxes representing three replicates were harvested and immediately frozen in liquid nitrogen for RNA isolation. Identical sampling procedures were followed for the slow germination variety (SLG), with the sampling done at 1, 3, and 5 DAI. For both genotypes, changes in seed weight were recorded at 1 and 3 DAI under normal and low temperature treatments, and the water uptake was measured as a fraction of dry seed weight^40^.

### RNA extraction, library construction, and sequencing

The total RNA was extracted using TRIzol reagent. The integrity of RNA was verified by RNase-free agarose gel electrophoresis. RNA purity and concentration were quantified using Nanodrop 2000 (Thermo, USA) and 2100 Bioanalyzer (Agilent Technologies, Santa Clara, CA). The high-quality RNA, with three biological replicates for each treatment, was enriched using Oligo(dT) beads. The enriched mRNA was cut into short fragments using fragmentation buffer and reverse-transcribed into cDNA with random primers. The second-strand cDNA was synthesized using DNA polymerase I, RNase H, dNTP, and buffer. Then, the cDNA fragments were purified using the QiaQuick PCR extraction kit, end-repaired, poly(A) added, and ligated to Illumina sequencing adapters. The ligation products were selected by size using agarose gel electrophoresis, PCR-amplified, and sequenced in 100-bp paired end reads using Illumina HiSeq2000.

### Transcriptome assembly and optimization

Reads obtained from the sequencers were filtered to remove reads containing adapters and those containing >10% unknown nucleotides (N), as well as the low-quality reads containing >50% low-quality bases. Next, the reads mapping to the ribosome RNA (rRNA) database were identified using the short reads alignment tool Bowtie2 and removed. The remaining high-quality clean reads of each sample were then mapped to the reference genome using TopHat2 (version2.0.3.12). The transcripts were reconstructed using Cufflinks software, allowing new genes and splice variants of known genes to be identified. Next, Cuffmerge was used to merge transcripts from different replicates of a group into a comprehensive set of transcripts, and the transcripts from multiple groups were then merged into a final, comprehensive set of transcripts for further downstream differential expression analysis. Principal component analysis (PCA) was performed using the prcomp function in R to determine the relationships between the sample transcripts.

### Normalization of gene expression levels and identification of differentially expressed genes (DEGs)

Gene abundances were quantified using RSEM software^41^. Gene expression levels were normalized using the fragments per kilobase of transcript per million mapped reads (FPKM) method. The edgeR package was used to identify DEGs across groups with a fold change ≥2 and a false discovery rate (FDR) < 0.05. DEGs were then subjected to enrichment analysis of GO functions and KEGG pathways defined by a hypergeometric test.

### Gene expression network construction and visualization

Weighted gene co-expression network analysis (WGCNA) is a systems biology method used to describe the correlation patterns among genes across multiple samples. This method finds clusters (modules) of highly correlated genes and related modules with external sample traits. Co-expression networks were constructed using WGCNA (v1.47) package in R. Before the analysis, genes with an average expression <1.5 FPKM among the samples, and those with proportion of no expression gene number >50% among the samples were filtered. After filtering, gene expression values were imported into WGCNA to construct co-expression modules using the automatic network construction function blockwiseModules with default settings, except that the power was 10, TOMType was unsigned, mergeCutHeight was 0.73, and Minimum ModuleSize was 50. To identify biologically or clinically significant modules, the module eigengenes was used to calculate the correlation coefficient for the samples. The intramodular connectivity of each gene was calculated and the genes with high degree tended to be hub genes, which might have important functions. The networks were visualized using R/igraph 1.0.0 package.

### Quantitative real-time PCR confirmation of the RNA-Seq data

Twenty genes were randomly selected in different expression pattern modules for quantitative real-time PCR with three biological replicates. cDNA was synthesized from 3 µg total RNA using the TUREscript 1st Stand cDNA SYNTHESIS Kit (Aidlab, China), according to the manufacturer’s protocol. The primers used for these experiments are listed in Additional file 2. PCR was set up in 96-well Hard-Shell PCR plates with 0.2 μM primers using SYBR® Green Supermix (BDI, German) in a reaction volume of 5 μL. The reaction conditions were as follows: denaturation at 95 °C for 3 min, 40 cycles of 95 °C for 10 s and 58 °C for 30 s, heating from 60 to 95 °C at a rate of 1 °C per 4 s for melting curve analysis. Two internal reference genes UBC9 and YLS8 were designed to normalize the relative gene expression levels using the 2^△△Ct^ method^42,43^.

## Results

### Variation in germination speed under low temperature and its relationship with seed components

**Figure 1:**
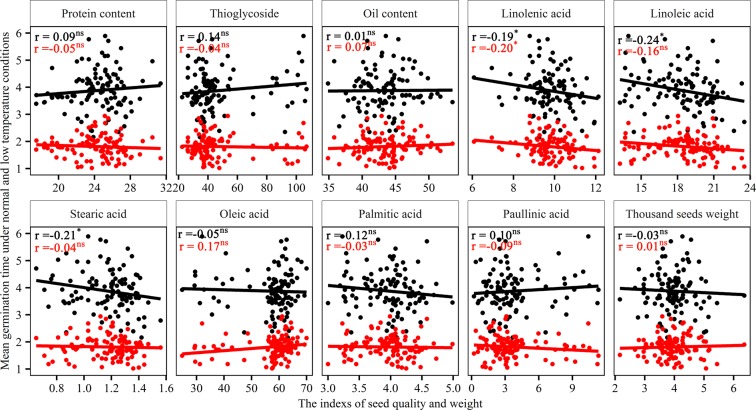
The correlation between mean germination time (MGT) and seed quality indexes of rapeseed genotypes (*Brassica napus* L.) under low temperature (15/10 °C) and normal temperature (25/20 °C). The black and red dots represent the data under low temperature and normal temperature, respectively. Seed quality indexes include linoleic acid (%), linolenic acid (%), oil content (%), oleic acid (%), palmitic acid (%), paullinic acid (%), protein content (%), stearic acid (%), thioglycoside (μmol/g) and thousand seed weight (g). “*” represents have significant difference at *P* < 0.05 level. “ns” represents no significant difference at *P* < 0.05 level.

A germination experiment was conducted under low temperature (15/10 °C) and control (25/20 °C) conditions with more than 100 genotypes of rapeseed (*Brassica napus* L.). The data of MGT of different genotypes and their seed components were provided in Additional file 1. The results showed that there was a significant variation in germination speed under low temperature stress, and the MGT ranged from 2.16 to 5.89 d for rapeseed. Analysis of the seed quality revealed significant variation in seed components and size. The total oil content in seed ranged from 34.8% to 51.7%. The stored protein content ranged from 18.8% to 31.4%, and the 1000-seed weight ranged from 2.2 to 6.3 g for these genotypes. There was no significant correlation between the germination speed and seed quality indexes, except for linolenic acid, linoleic acid and stearic acid under low temperature condition and linolenic acid under normal temperature condition (Fig. 1). These results suggested that the seed components and size were not the main factors determining the germination speed of rapeseed.

### Germination characteristics of the fast and slow germination genotypes responding to low temperature stress

**Figure 2:**
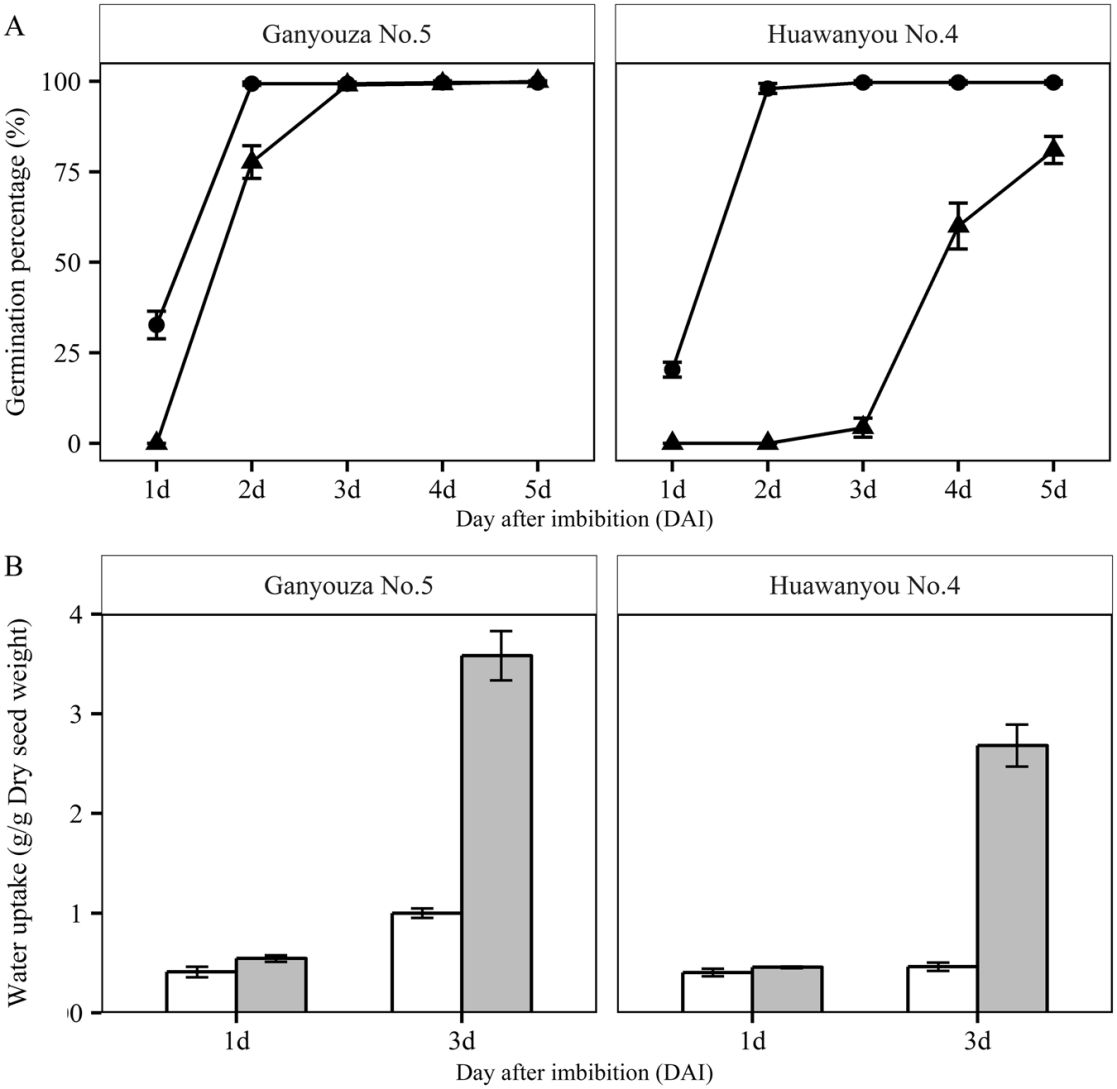
The germination progresses and imbibition condition of Ganyouza No. 5 and Huawanyou No. 4 under normal and low temperature treatments. (**A)** The cumulative germination percentage after imbibition (●, normal temperature; ▲, low temperature). (**B**) The water uptake at 1 and 3 days after imbibition (The white bar, low temperature; grey bar, normal temperature). Values are mean ± SD for three replications.

The germination progresses of Ganyouza No. 5 and Huawanyou No. 4 under low temperature and control conditions were showed in Fig. 2. Under normal temperature condition, the germination processes of these two genotypes were almost synchronous, while a significant difference in the germination speed was observed under low temperature stress. No germination was observed at 1 DAI under low temperature (Fig. 2A). The germination of Ganyouza No. 5 occurred at 2 DAI and most seeds completed germination at 3 DAI under low temperature (Fig. 2A). For Huawanyou No. 4, germination at low temperature occurred at 3 DAI and 75% of seeds finished germination at 5 DAI. Under normal temperature conditions, the mean germination time for Ganyouza No. 5 and Huawanyou No. 4 were 1.68 and 1.85 d, respectively. The germination speed of Ganyouza No. 5 remained fast under low temperature condition, with a mean germination time of 2.91 d; while that of Huawanyou No. 4 decreased, with a mean germination time of 4.59 d. The water uptake was higher under normal temperature than under low temperature treatment, especially at 3 DAI; The fast germination speed genotype Ganyouza No. 5 also had a higher imbibition speed compared to Huawanyou No. 4 (Fig. 2B).

### RNA sequence quality

**Figure 3:**
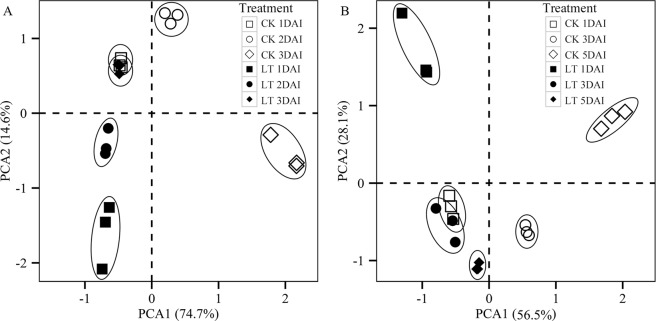
Principal component analysis (PCA) of transcript profiles during seed germination. (**A**), FAG; (**B**), SLG. The FAG and SLG represent genotypes of Ganyouza No. 5 and Huawanyou No. 4, respectively. The CK and LT indicate germination treatment under the low temperature and normal temperature, respectively. The DAI means the day after imbibition of seed germination.

Two representative genotypes, FAG and SLG were used for transcriptome analysis. Approximately 143.4 G raw data were generated from 36 samples at different developmental stages though RNA sequencing (Additional file 3 and Additional file 4). The raw sequence data have been submitted to the NCBI Short Read Archive under accession number SRP111037. After filtering and trimming of raw reads, 1 016 103 042 high-quality reads (average Q30 > 92.7%) were used for further analysis. The reference genome of *Brassica napus* L. was predicted to have 101 040 genes, while 71 317 and 74 069 genes were found to be mapped to the reference genome during the germination process for FAG and SLG, respectively. Furthermore, 3881 new genes were discovered across the 36 samples. The PCA showed that the percentage of total variation accounted for by the first and second principal components were 89.3 and 84.6 for FAG and SLG, respectively (Fig. 3). The three biological repeats of experimental data were generally consistent, as seen in the distribution of these samples (Fig. 3).

### Weight gene co-expression network analysis

**Figure 4:**
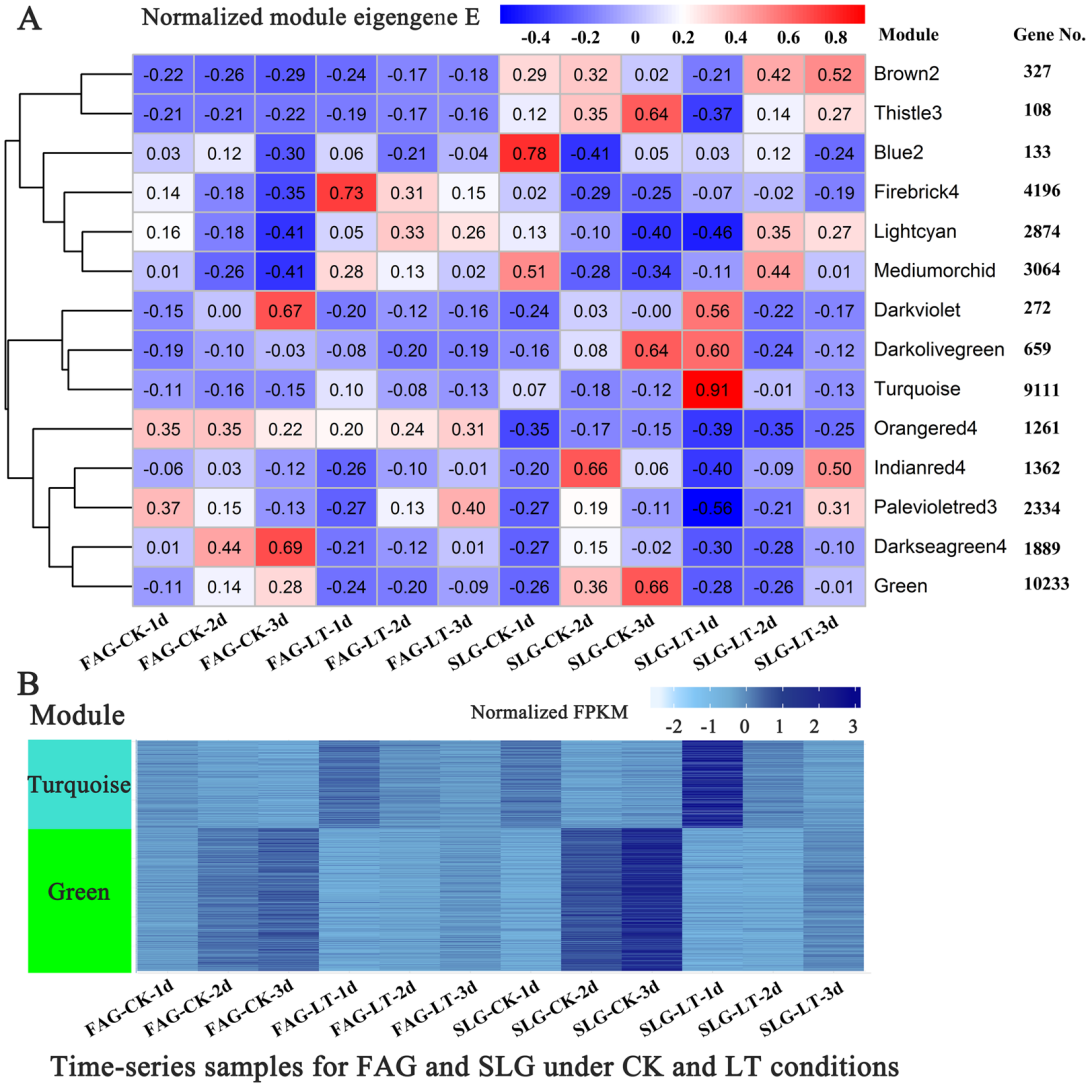
The gene expression pattern of different modules. (**A**) The module eigengene E distribution of co-expressed modules for time-series samples of FAG and SLG under CK and LT conditions. The number of gene distributed in each module is indicated on the right. The modules relationships have been showed by K-means clustering. The color of each cell at the row-column intersection indicates module eigengene E, which is defined as the first principal component and can be consider as a representative of the module’s genes expression level for a specific sample. The high expression level between a specific module and samples is indicated by dark red. (**B**) Heat map showing the normalized FPKM of each gene from the turquoise module and green module. The FAG and SLG represent genotypes of Ganyouza No. 5 and Huawanyou No. 4, respectively. The CK and LT represent normal and low temperature treatments, respectively.

Correlation networks are being increasingly used in bioinformatics applications. The weighted gene co-expression network analysis (WGCNA) is a systems biology method used to describe the correlation patterns among genes across transcriptomic libraries, and to screen candidate genes related to phenotypic traits. In the present study, WGCNA was based on the temporal transcript profiling of three time points during the germination process for fast and slow germination speed genotypes under low and normal temperature conditions. In total, 37 823 genes were clustered into 14 modules by hierarchical clustering with a mergeCutHeight of 0.73, and branches in the hierarchical clustering dendrograms corresponded to modules with a different color (Additional file 5). Genes within the same module were highly correlated with each another (Additional file 6). The relationship between modules and samples is showed in Fig. 4A. The number of genes in each categories is presented on the right. Most genes were distributed in the green (10 233) and turquoise (9111) modules. The module eigengene calculated as the first principal component of a given module could be consider as a representive of gene expression levels among these samples in a module. The dark red of cell at the row-column intersection indicated a high degree of correlation between a specific module and sample. The genes in turquoise module had a high expression at 1 DAI for the SLG under low temperature stress, whereas the genes in palevioletred3 module had a low expression under the same condition (Fig. 4A). As showed in Fig. 4B, the transcript abundance of most genes in the green module continued to up-regulate over time for the FAG and SLG under both low and normal temperature conditions during the germination process, whereas that in the turquoise module showed the opposite expression pattern. The gene expression patterns in green and turquoise modules tended to show positive and negative synchronization with the germination process, respectively. Meanwhile, more than half of the DEGs were distributed in these modules. It could assume to a large extent that the genes clustered in the green and turquoise modules played a fundamental role in seed germination in both FAG and SLG.

The genes distributed in the green (continuous up-regulation) and turquoise (continuous down-regulation) modules were separately annotated and classified into three groups by GO enrichment analysis, which were biological process, molecular function, and cellular component (Table 1). The genes in the green and turquoise modules displayed a different trend in the GO term. Under biological process, secondary metabolic process was the most significantly abundant GO term for the green module, followed by sulfur compound metabolism, and small molecular metabolism; The GO term response to organonitrogen compound were significantly enriched for turquoise module, followed by response to acid chemical process and protein modification process. Under cellular component, the major categories including plastid thylakoid, organelle sub-compartment, and chloroplast thylakoid were significantly enriched for the green module; the major categories including lipid particle, nucleus and nucleoplasm were significantly enriched for turquoise module. In molecular function, gene-encoding proteins that participate in oxidoreductase activity, hydrolase activity and tetrapyrrole binding were highly represented in the green module; nucleic acid binding, organic cyclic compound binding and ubiquitin-like protein transferase activity were highly presented for turquoise module (Table 1).

**Table 1:** The top 10 GO terms in biological process, cellular component and molecular function catogories in green and turquoise modules.

Class	Module Green		Module Turquoise	
	GO ID	Description	GO ID	Description
Cellular Component	GO:0031976	plastid thylakoid	GO:0005811	lipid particle
	GO:0031984	organelle subcompartment	GO:0005634	nucleus
	GO:0009534	chloroplast thylakoid	GO:0005654	nucleoplasm
	GO:0034357	photosynthetic membrane	GO:0043231	intracellular membrane-bounded organelle
	GO:0009521	photosystem	GO:0043227	membrane-bounded organelle
	GO:0009532	plastid stroma	GO:0030662	coated vesicle membrane
	GO:0009526	plastid envelope	GO:1990234	transferase complex
	GO:0005576	extracellular region	GO:0000785	chromatin
	GO:0009570	chloroplast stroma	GO:0030135	coated vesicle
	GO:0031224	intrinsic component of membrane	GO:0030659	cytoplasmic vesicle membrane
Molecular Function	GO:0016491	oxidoreductase activity	GO:0003676	nucleic acid binding
	GO:0016798	hydrolase activity	GO:0097159	organic cyclic compound binding
	GO:0046906	tetrapyrrole binding	GO:0019787	ubiquitin-like protein transferase activity
	GO:0004553	hydrolase activity, hydrolyzing O-glycosyl compounds	GO:0016887	ATPase activity
	GO:0003824	catalytic activity	GO:0046914	transition metal ion binding
	GO:0016209	antioxidant activity	GO:0019840	isoprenoid binding
	GO:0015103	inorganic anion transmembrane transporter activity	GO:1901363	heterocyclic compound binding
	GO:0008092	cytoskeletal protein binding	GO:0001071	nucleic acid binding transcription factor activity
	GO:0015631	tubulin binding	GO:0008375	acetylglucosaminyltransferase activity
	GO:0005372	water transmembrane transporter activity	GO:0004721	phosphoprotein phosphatase activity
Biological Progress	GO:0044550	secondary metabolite biosynthetic process	GO:0010243	response to organonitrogen compound
	GO:0006790	sulfur compound metabolic process	GO:0001101	response to acid chemical
	GO:0044283	small molecule biosynthetic process	GO:0036211	protein modification process
	GO:0044711	single-organism biosynthetic	GO:0006464	cellular protein modification process
	GO:0015979	photosynthesis	GO:0044260	cellular macromolecule metabolic process
	GO:0006811	ion transport	GO:0050794	regulation of cellular process
	GO:0006812	cation transport	GO:0044267	cellular protein metabolic process
	GO:0016144	S-glycoside biosynthetic	GO:0007165	signal transduction
	GO:0019758	glycosinolate biosynthetic	GO:0009719	response to endogenous stimulus
	GO:0009812	flavonoid metabolic	GO:0023052	signaling

**Figure 5:**
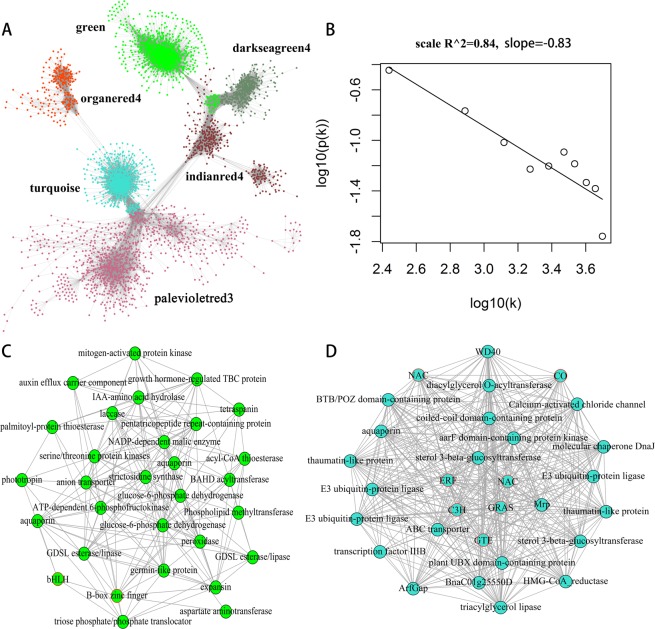
Properties and topologies of gene co-expression networks. (**A**) The entire co-expression network constructed across different modules. The dots represent genes and undirected lines represent significant transcriptional interactions between the genes (**B**) The log-log plot of whole network connectivity distribution. Linear fitting with a high R^2 indicates a good scale free topology of network. (**C**) The correlation network of hub genes (green color) with connectivity ranking in top 30 for green module. (**D**) The correlation network of hub genes (turquoise color) with connectivity ranking in top 30 for turquoise module. Trancription factors are represented by red circle. For each gene, the connectivity is defined as the sum of collection strengths with the other network genes.

A weighted gene co-expression network was constructed based on the connectivity of genes across different modules (Fig. 5A). This graph showed a visualization of the interactions of genes according to topological overlap, and the clusters with different colors indicated different expression patterns among the time-series samples. The logarithm of the entire network connectivity corresponding to the connectivity frequency distribution showed a linear relationship, which was referred to as approximately scale-free topology (Fig. 5B). The hub genes in scale-free topology network tended to have high degree inside a co-expression module. In the green module, five hub genes were found to participate in phytohormone regulation and signal transduction, including growth hormone-regulated TBC protein (*BnaA03g30230D*), auxin efflux carrier component (*BnaC06g24400D*), IAA-amino acid hydrolase (*BnaC09g33210D*), serine/threonine protein kinases (*BnaA05g31460D*), mitogen-activated protein kinase (*BnaA01g21880D*), and phototropin (*BnaA06g19860D*). Two glucose-6-phosphate dehydrogenase genes (*BnaCnng48180D* and *BnaCnng78750D*) and one 6-phosphofructokinase gene (*BnaC01g09280D*) acted as hub genes in the green module, which are the rate-limiting enzymes in the pentose phosphate pathway and glycolysis, respectively. Two GDSL esterase/lipase proteins (*BnaA07g08280D* and *BnaA04g04900D*) and acyl-CoA thioesterase (*BnaC02g27480D*), which are a very large family of lipolytic enzymes, were identified as hub genes (Fig. 5C). Interestingly, six of the top 30 hub genes in the turquoise module encoded transcription factors, including NAC (*BnaC01g23890D* and *BnaA09g04810D*), GRAS (*BnaC08g02280D*), zinc finger protein (*BnaC04g32370D* and *BnaA08g18650D*), and ERF (*BnaC03g49530D*). Two hub genes, E3 ubiquitin-protein ligase (*BnaC01g04040D* and *BnaC09g38770D*), participated in ubiquitin-mediated proteolysis in the turquoise network (Fig. 5D).

**Figure 6:**
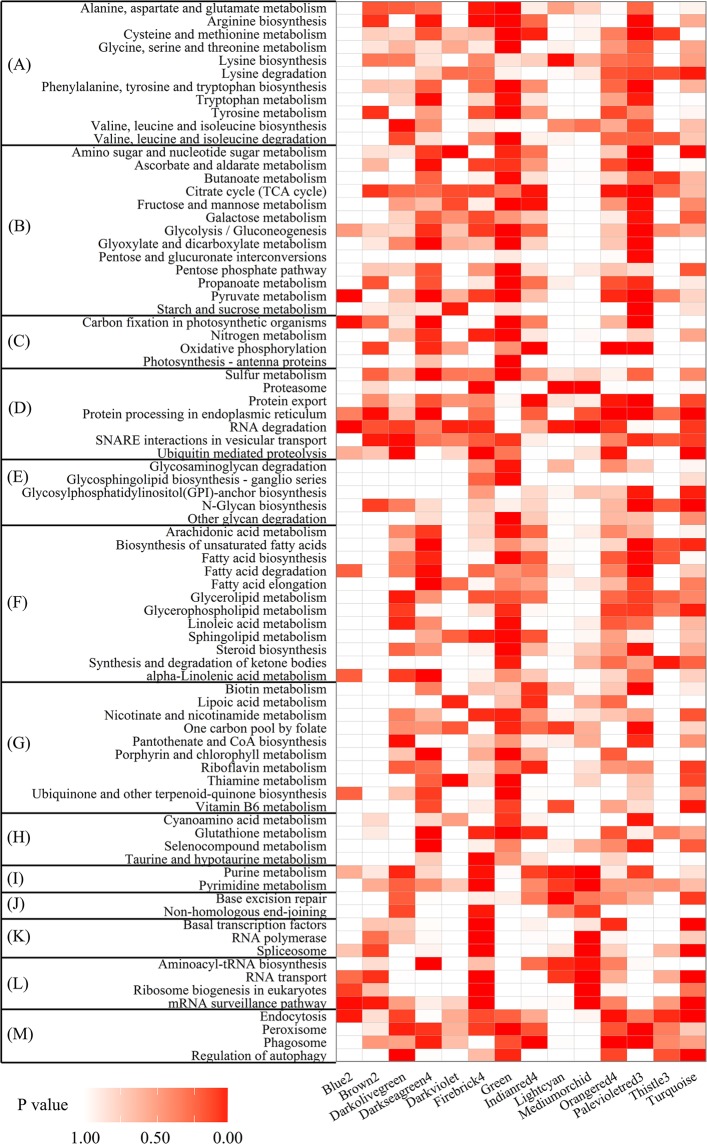
The distributions of function for different expressed gene in all the clustered modules by KEGG enrichment analysis. (**A**) Amino acid metabolism. (**B**) Carbohydrate metabolism. (**C**) Energy metabolism. (**D**) Folding, sorting and degradation. (**E**) Glycan biosynthesis and metabolism. (**F**) Lipid metabolism. (**G**) Metabolism of cofactors and vitamins. (**H**) Metabolism of other amino acids. (**I**) Nucleotide metabolism. (**J**) Replication and repair. (**K**) Transcription. (**L**) Translation. (**M**) Transport and catabolism. On the x-axis, the different colors represent modules with different expression pattern.

KEGG pathway enrichment analysis was used to determine the functional significance of genes in each module (Fig. 6). The results indicated that 10 590 genes were annotated in 13 categories. A part of genes in green module were enriched in pentose phosphate pathway, a part of genes in green and palevioletred3 modules were enriched in glycolysis pathway, and a part of genes in indianred4, orangered4, and palevioletred3 modules were enriched in citrate cycle pathway; all these genes played important roles in carbohydrate metabolism and energy production during germination. As rapeseed seeds are rich in oil, the degradation and mobilization of oil bodies in cotyledons have been activated after imbibition. A part of genes in darkseagreen4, green and palevioletred3 modules were enriched in lipid metabolism during germination, including arachidonic acid, glycerolipid, glycerophospholipid, linoleic acid, and sphingolipid metabolism. Expressed genes in the green and palevioletred3 modules are significantly enriched in glyoxylate and dicarboxylate metabolism, which is involved in the biosynthesis of carbohydrates from fatty acids. Expressed genes in the firebrick4, lightcyan, mediumorchid, and turquoise modules contributed mostly to nucleotide metabolism, replication and repair, transcription, and translation.

### DEGs under low temperature for FAG and LSG

**Figure 7:**
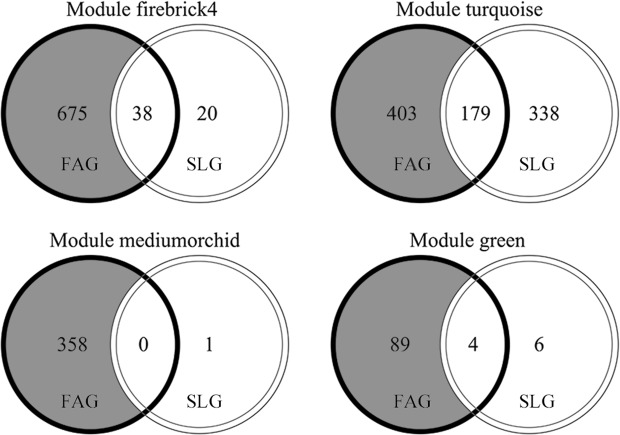
Venn diagram of continued up-regulated genes under low temperature stress on different modules for FAG and SLG. The black circle and the white circle represent the continued up-regulated genes under low temperature stress for FAG and SLG. The grey section represents the specific up-regulated genes in FAG, which excludes the continued up-regulated genes in SLG. The FAG and SLG represent genotypes of Ganyouza No. 5 and Huawanyou No. 4, respectively.

Pairwise comparison of expressed genes under low and normal temperature conditions revealed that 1993 DEGs continued to be upregulated and 2962 DEGs continued to be downregulated for FAG, while 674 DEGs continued to be upregulated and 1432 DEGs continued to be downregulated for SLG (Additional file 7 and Additional file 8). For FAG, a greater number of genes responded to low temperature stress compared with SLG. The distribution of continued upregulated genes of FAG and SLG in modules were showed in Fig. 7. For FAG, most upregulated DEGs were distributed in the firebrick4 module, followed by the turquoise and mediumorchid modules. For SLG, most of the upregulated DEGs were distributed in the turquoise module (Fig. 7). The grey section in Fig. 7 represents the specific upregulated genes (excluding the DEGs upregulated in SLG), which could provide information to explain the fast germination for FAG.

The specific upregulated DEGs under low temperature conditions for FAG were subjected to KEGG pathway enrichment analysis. Only 25.1% of these DEGs were annotated in the KEGG database. The top 20 KEGG pathways with the highest representation of these DEGs are showed in Table 2. RNA polymerase, spliceosome, RNA transport, and ribosome biogenesis in eukaryotes pathways related to transcription and translation were significantly enriched. Meanwhile mRNA surveillance and RNA degradation pathways were enriched to control the quality of mRNA under low temperature conditions for FAG. Twelve chaperone protein genes continued to be upregulated, which contributed to the proper assembling of proteins. Defense systems, including plant-pathogen interactions, ascorbate and aldarate metabolism, and glutathione metabolism were also found to participate in the response to low temperature in FAG.

**Table 2:** The top 20 of KEGG pathways with high representation for the specific up-regulated DEGs of FAG under low temperature stress.

Pathways	All modules	Firebrick4	Mediumorchid	Turquoise	Green
	(% of 383)	(% of 166)	(% of 106)	(% of 82)	(% of 19)
Ribosome biogenesis in eukaryotes	102 (26.63%)	36 (21.69%)	51 (48.11%)	10 (12.20%)	5 (26.32%)
Spliceosome	61 (15.93%)	26 (15.66%)	11 (10.38%)	19 (23.17%)	1 (5.26%)
RNA transport	33 (8.62%)	11 (6.63%)	12 (11.32%)	8 (9.76%)	2 (10.53%)
Pyrimidine metabolism	28 (7.31%)	11 (6.63%)	14 (13.21%)	2 (2.44%)	0 (0.00%)
Purine metabolism	27 (7.05%)	9 (5.42%)	14 (13.21%)	4 (4.88%)	0 (0.00%)
mRNA surveillance pathway	26 (6.79)	13 (7.83%)	7 (6.60%)	6 (7.32%)	0 (0.00%)
RNA degradation	22 (5.74%)	8 (4.82%)	9 (8.49%)	2 (2.44%)	2 (10.53%)
RNA polymerase	21 (5.48%)	7 (4.22)	12 (11.32%)	2 (2.44%)	0 (0.00%)
Plant hormone signal transduction	19 (4.96%)	10 (6.02%)	4 (3.77%)	4 (4.88%)	1 (5.26%)
Homologous recombination	13 (3.39%)	9 (5.42)	1 (0.94%)	1 (1.22%)	2 (10.53%)
Protein processing in endoplasmic reticulum	12 (3.13%)	4 (2.41%)	0 (0.00%)	6 (7.32%)	1 (5.26%)
Mismatch repair	9 (2.35%)	5 (3.01%)	1 (0.94%)	1 (1.22%)	2 (10.53%)
Nucleotide excision repair	9 (2.35%)	4 (2.41%	1 (0.94%)	1 (1.22%)	3 (15.79%)
Ribosome	9 (2.35%)	5 (3.01%)	1 (0.94%)	3 (3.66%)	0 (0.00%)
DNA replication	8 (2.09%)	4 (2.41%	1 (0.94%)	1 (1.22%)	2 (10.53%)
Plant-pathogen interaction	8 (2.09%)	4 (2.41%	0 (0.00%)	2 (2.44%)	2 (10.53%)
Ascorbate and aldarate metabolism	7 (1.83%)	5 (3.01%)	2 (1.89%)	0 (0.00%)	0 (0.00%)
Glutathione metabolism	7 (1.83%)	6 (3.61%)	0 (0.00%)	1 (1.22%)	0 (0.00%)
Phosphatidylinositol signaling system	7 (1.83%)	2 (1.20%)	0 (0.00%)	3 (3.66%)	2 (10.53%)
ABC transporters	6 (1.57%)	0 (0.00%)	4 (3.77%)	2 (2.44%)	0 (0.00%)

**Figure 8:**
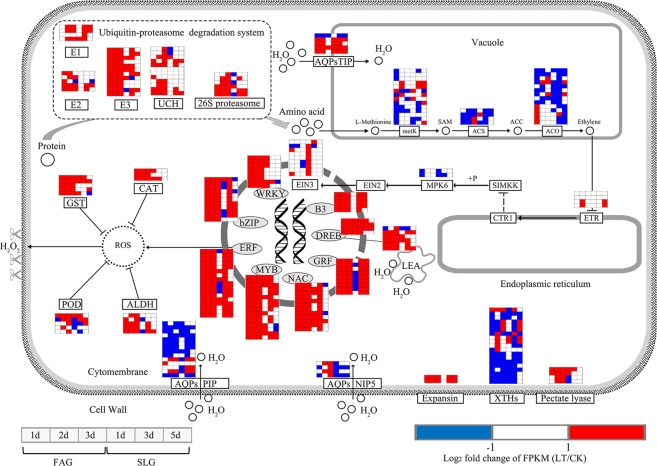
Comparative transcriptional change of genes responding to low temperature stress for these two genotypes in germination process. The oval represents transcriptional factors and the rectangle represents enzyme or proteins. For enzyme reactions, the arrows between two metabolites represent the direction of catalytic reactions. For aquaporin (AQPs), the arrows cross the rectangle indicate the direction of water transport. The FAG and SLG represent genotypes of Ganyouza No. 5 and Huawanyou No. 4, respectively.

Although a large proportion of upregulated DEGs under low temperature stress were not enriched in KEGG pathway, they may also played an important role in the fast germination of FAG. Strikingly, 113 genes among the 1551 specific upregulated DEGs encoded transcription factors, including WRKY, bZIP, EFR, MYB, B3, DREB, NAC, and ERF (Fig. 8). Genes related to cell-wall loosening and remodeling were markedly expressed during germination, in which expansin (*BnaCnng40260D* and *BnaA09g29240D*), xyloglucan endotransglucosylase/hydrolase (*BnaCnng23300D*), and pectate lyase (*BnaC05g43400D* and *BnaA05g28930D*) continued to be upregulated in FAG under low temperature stress during germination (Fig. 8). Three classes of aquaporin (PIP, NIP5, and TIP) were differentially expressed during seed germination (Fig. 8). Four aquaporin proteins (AQPs) TIP genes (*BnaA06g12030D*, *BnaA09g44820D*, *BnaC05g13770D*, and *BnaC08g37510D*), located in the vacuole membrane, were upregulated, whereas most of the AQPs PIP genes were downregulated under low temperature conditions for these two genotypes. The AQPs NIP5 genes (*BnaA03g24370D* and *BnaC03g28980D*) were upregulated under low temperature conditions in FAG. The late embryogenesis abundant (LEA) protein genes also involved in the germination process, in which *BnaCnng65680D*, *BnaA07g10370D*, *BnaC02g31000D*, *BnaA02g22630D* and *BnaA10g01720D* were continued to upregulated under low temperature stress for FAG. The genes encoding the critical enzymes (ACS and ACO) for ethylene biosynthesis were detected, but most of them were downregulated under low temperature conditions, suggesting that slow ethylene production might be one of the main factors contributing to slow germination speed under low temperature stress. Additionally, 17 ethylene-responsive transcription factors (ERF) continued to be upregulated under low temperature stress for FAG. The ERF could mediate the production of reactive oxide species (ROS). Genes related to ROS scavenging were also upregulated under low temperature conditions in FAG (Fig. 8), including glutathione S-transferase (GST), catalase (CAT), aldehyde dehydrogenase (ALDH), and peroxidase (POD).

Many protein families among the specific upregulated DEGs in FAG were detected, including WD repeat-containing protein (six in the mediumorchid module), pentatricopeptide repeat-containing protein (seven in the firebrick4 module and seven in the mediumorchid module), high mobility group B protein (five in the firebrick4 module), cytochrome P450 (six in the firebrick4 module), and coiled-coil domain-containing protein (four in the turquoise module). Furthermore, two programmed cell-death protein genes (*BnaA09g00670D* and *BnaC03g49430D*) expressed with a higher transcription level during germination in the fast germinating genotypes under low temperature conditions, compared with the slow germinating genotype. These might contribute to the fast FAG germination speed observed under low temperature stress; however, their specific functions during germination require further study.

### RNA sequencing validation by qRT-PCR

**Figure 9:**
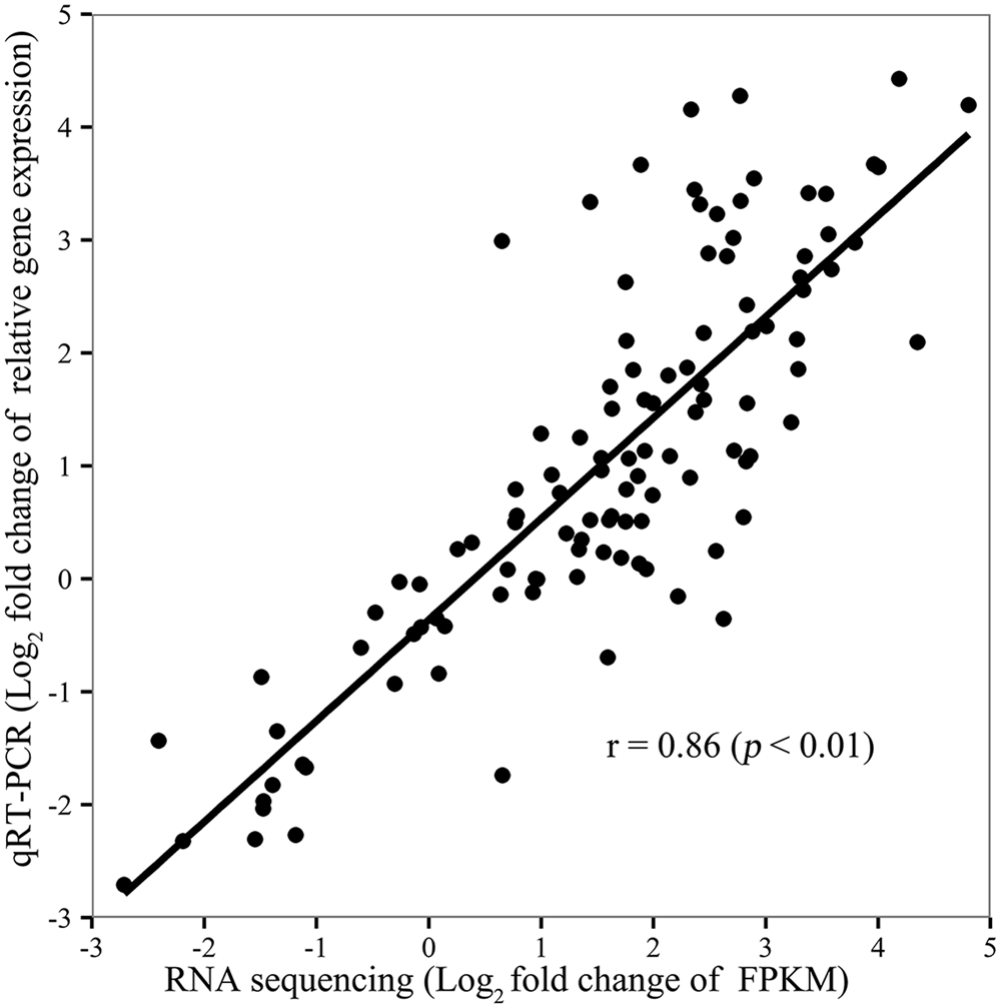
Correlation between qRT-PCR and RNA sequencing for twenty selected genes from green, turquoise, mediumorchid and firebrick4 modules. Relative gene expression are means of three replications. Each point represents fold change of expression level at low temperature comparing with that at normal temperature for each sample.

To confirm the accuracy and reproducibility of the transcriptome analysis, 20 genes from the green, turquoise, mediumorchid, and firebrick4 modules were randomly selected for real-time quantitative reverse transcription PCR (qRT-PCR) validation. Primers of these candidate genes showed in Additional file 2. Linear regression analysis of the fold-change in gene expression ratios under low and normal temperature conditions between RNA-seq and qRT-PCR data revealed a positive correlation with high significance (*p* < 0.01, Fig. 9), which suggested that the expression results generated by RNA sequencing were reliable.

## Discussion

Germination speed differed among rapeseed (*Brassica napus* L.) genotypes, especially under low temperature stress. Fast germination speed is a derived trait, which evolved as an adaptation to adverse environments, including low temperature, arid, saline, or floodplain conditions^44–46^. There is significant variation in seed components and size, which have been reported to be relevant for seed vigor^47,48^. However, these indices did not determine the germination speed of rapeseed seeds in our study. Seed germination is a dynamic and ordered process involving several biochemical pathways^49,50^. Differential regulation of transcriptome in resistance to low temperature stress likely plays an important role in producing fast germination speed. Heterotrophic metabolism was a crucial step contributing to seed germination^51^, and the effective degradation and mobilization of predominant reserves was essential for the success of germination^52^. Early protein biosynthesis during germination depends on the free amino acids released by the storage proteins. Ubiquitin-protein ligase and 26S proteasome are synthesized de novo, and are synergistically activated to promote the mobilization of reserve protein during the seed germination process^53^. In present study, the expression of most 26S proteasome and ubiquitin-protein ligase genes showed a decreasing tendency during the germination process under normal temperature conditions for both slow and fast germination genotypes. Under low temperature stress, this expression of some 26S proteasome and ubiquitin-protein ligase genes increased in the fast germinating genotype after 1 DAI, whereas only a few of 26S proteasome and ubiquitin-protein ligase genes improved their expression after 3 DAI in the slow germinating genotype (Fig. 8). This expression pattern suggested that the fast germination speed genotype mobilized protein reserves more rapidly than the slow germination speed genotype under low temperature stress.

Low temperatures slowed down the rate of seed water uptake, and the fast germination speed genotype showed a higher imbibition speed under low temperature stress, compared to slow germination speed genotype. Previous studies have demonstrated that the accumulation of LEA proteins in embryos correlated with the acquisition of desiccation tolerance during seed maturation^54,55^. As the LEA proteins are strongly hydrophilic and possess a high degree of flexibility, they interact with a variety of cellular components, including other proteins, and contribute to stability either by sharing their hydration shell, or by using their own hydroxylated amino acids to serve as a replacement for water^54,56–58^. The LEA protein genes may also play an important role in the initial phase of seed imbibition. Under normal temperature condition, the expression of LEA protein genes decreased during germination, this being correlated with an increase of free water in seeds. For the fast germination genotype, the LEA protein genes maintained a higher expression under low temperature stress compared with normal temperature. This up-regulation of LEA protein genes under low temperature stress may help alleviate the low water absorption and increase the water-use efficiency, contributing to a fast and uniform germination with low water uptake. Three classes of aquaporin family genes expressed during germination to accelerate the process of water uptake and vacuole enlargement, wherein *BnaC04g08100D* and *BnaA06g12030D* acted as hub genes in green module and turquoise modules, respectively. Most aquaporin PIP genes were downregulated under low temperature stress, while some of the aquaporin NIP5 genes were upregulated in the fast germination speed genotype. The aquaporin NIP5 is located in the plasma membrane and transports boric acid under boron limitation^59,60^. The upregulated expression of aquaporin NIP5 under low temperature stress might contribute to water uptake during the seed germination process.

The fast germination speed genotype displayed rapid signal transduction and transcriptome regulation in response to low temperature stress. For fast germination speed genotype, a part of specific upregulated genes under low temperature enriched in the phosphatidylinositol signaling system. Compared with the slow germination speed genotype, genes encoding serine/threonine-protein kinase, casein kinase, and calmodulin were also upregulated in the fast germinating genotypes at low temperature, supporting that IP3/Ca2+ signal transduction pathway played an important role in abiotic stress^24,61^. The transcription factor families, including bZIP, bHLH, AP2/ERF, MYB, NAC, GRF, zinc-finger, WRKY, HSF, and Trihelix, were upregulated to response to low temperature stress and participated in the transcriptional regulation in fast germination speed genotypes. Consistent with this, chilling-tolerance showed to be related to the upregulation of shock proteins and heat shock transcription factor genes^62,63^. Twelve chaperone protein genes were upregulated under low temperature stress in the fast germinating genotypes, which had surveillance role, promoting efficient protein folding and ensuring protein homeostasis^64^.

Ethylene-responsive transcription factors (ERFs) are a specific class of plant transcriptional regulators that mediate ethylene-dependent gene expression by binding to the GCC motif found in the promoter region of ethylene-regulated genes^65–67^. Ethylene production is correlated with time to radicle protrusion in a number of species, including *Lactuca sativa*^68^, *Nicotiana tabacum*^69^, *Pisum sativum*^70^, *Solanum lycopersicum*^65^, and *Arabidopsis thaliana*^71^. Two 1-aminocyclopropane-1-carboxylate synthase (ACC) genes and 14 1-aminocyclopropane-1-carboxylate oxidase (ACO) genes were distributed in the green module; these enzymes mediate the rate-limiting step in ethylene biosynthesis and control ethylene evolution during seed germination^72,73^. Expressions of these ethylene-related enzyme genes were lower under low temperature regardless of the germination speed genotype, while the expression of many ERFs in the fast germination genotype continued to be upregulated under low temperature stress during germination compared with the response under normal temperature. Activation of the ERF-related signal pathway might accelerate the transition from a quiescent state to a highly active metabolic state under low temperature stress by increased reactive oxygen species (ROS) content, especially H_2_O_2_. ROS are also known to act directly by non-enzyme scission of cell-wall polymers leading to cell-wall loosening and extension of embryos during germination^74,75^, as well as the weakening of surrounding tissues during fruit ripening^76^. H_2_O_2_ acts downstream of ethylene, and the ethylene/H_2_O_2_ mediated signal pathway plays an important role in programmed cell death^77,78^. ROS scavenging system genes, including CAT, ALDH, and GST were also upregulated to alleviate the ROC toxicity in cells and organelles in the fast germinating genotype under low temperature stress. The ability of seeds to germinate might be related to their capacity to regulate ROS homeostasis, which results from the balance between ROS producing and ROS-scavenging processes^79^. Biochemical enzymes mediating cell-wall remodelling are specifically expressed during germination^72,80,81^, with xyloglucan endotransglucosylase/hydrolase and pectate lease genes upregulated in the fast germination speed genotypes under low temperatures, compared with the response under normal temperature.

## Conclusions

There was a significant variation of germination speed among genotypes of rapeseed. However, seed components and size do not determine seed germination speed. Transcription factors, including WRKY, bZIP, EFR, MYB, B3, DREB, NAC, and ERF, are associated with low temperature stress in the fast germination genotype. The ethylene/H_2_O_2_-mediated signal pathway plays an important role in cell wall loosening and extension of embryos during germination for FAG. The ROS-scavenging system related genes were up regulated to alleviate the toxicity caused by ethylene/H_2_O_2_. The hub genes related to low temperature stress and their linking changes in the expression have been revealed on a global differentially expressed gene scale by co-expression network, but molecular mechanisms of gene-gene interaction, including direct and indirect relationships, still need a further research. These findings should be useful for molecular assisted screening and breeding of fast germination speed genotypes for rapeseed.

## Supplementary information

Additional file
