# Supplementary material for: Associating transcriptional regulation for rapid germination of rapeseed (Brassica napus L.) under low temperature stress through weighted gene co-expression network analysis
Source: Sci Rep. 2019 Jan 11;9:55. doi: 10.1038/s41598-018-37099-0 (PMC6329770; doi:10.1038/s41598-018-37099-0)

Associating transcriptional regulation for rapid germination of rapeseed (*Brassica napus* L.) under low temperature stress through weighted gene co-expression network analysis

Tao Luo, Mengzhu Xian, Chen Zhang, Chunni Zhang, Liyong Hu and Zhenghua Xu*

Institutional addresses:

MOA Key Laboratory of Crop Ecophysiology and Farming System in the Middle Reaches of the Yangtze River/College of Plant Science and Technology, Huazhong Agricultural University, Wuhan 430070, China.

Email addresses:

Tao Luo: luotao28@webmail.hzau.edu.cn

Mengzhu Xian, xianmengzhu_misty@126.com

Chen Zhang: 997542363@qq.com

Chunni Zhang: 1030591475@qq.com

Liyong Hu: liyonghu@mail.hzau.edu.cn

***For correspondence**: xzh@mail.hzau.edu.cn

Additional file 1: The mean germination time of different genotypes under normal and low temperature conditions and their seed components. The MGT represents the index of mean germination time. The LT and CK indicate germination treatment under the low temperature and normal temperature, respectively. The seed quality indexes including linoleic acid (%), linolenic acid (%), oil content (%), oleic acid (%), palmitic acid (%), paullinic acid (%), protein content (%), stearic acid (%) and thioglycoside (μmol/g) for screened genotypes was were analyzed with intact seeds weighted about 8 g by using a Near Infrared Reflectance Spectroscopy System.

Genotypes	MGT		Protein content	Thioglycoside	Oil content	Linolenic acid	Linoleic acid	Stearic acid	Oleic acid	Palmitic acid	Paullinic acid	1000 seeds weight
	LT	CK	%	μmol/g	%	%	%	%	%	%	%	g
1321	3.61	1.96	19.29	31.57	47.75	8.10	21.05	1.31	57.07	3.45	4.04	3.43
01188	3.63	1.80	19.95	22.40	50.25	9.68	22.15	1.38	62.42	3.68	2.25	3.09
10-804	3.48	2.02	21.44	47.83	41.95	9.75	18.49	1.15	56.28	3.91	3.54	4.99
2011-7103	3.69	1.41	19.77	47.17	46.03	9.70	20.29	1.27	58.76	4.29	3.89	4.05
2012-8355	3.54	1.98	23.16	39.34	42.68	9.92	18.82	1.17	57.27	3.98	3.61	3.92
2012-9354	3.73	1.94	19.83	31.89	50.40	9.71	20.22	1.39	58.62	4.69	3.96	4.29
9F087	3.63	1.78	20.42	33.92	48.61	7.30	16.43	1.34	63.22	4.53	3.11	3.51
Chuangyou 40	4.23	2.01	29.38	42.89	39.13	10.46	18.09	1.26	62.12	4.03	3.93	5.06
CY12GJ-1	3.39	1.70	17.52	38.75	46.29	10.76	20.41	1.27	60.12	4.31	2.91	4.74
CY12PXW-9	3.71	2.03	17.54	35.97	47.14	10.96	17.78	1.30	59.26	4.39	3.98	3.49
CY15PXW-31	3.45	1.92	18.47	40.45	53.55	9.44	20.61	1.47	58.85	4.99	2.03	3.04
Dadi 95	3.68	1.57	21.79	31.24	48.77	10.71	17.09	1.17	66.89	3.25	0.22	3.83
Dadi 99	3.65	1.65	19.25	31.95	51.74	12.13	19.56	1.20	63.55	3.79	2.14	4.07
Defuyou 508	2.35	1.33	26.67	92.31	41.07	9.50	14.39	0.87	47.35	3.37	6.61	5.00
Deheyou 898	5.89	2.68	25.20	104.93	43.54	8.79	13.94	1.03	32.04	3.23	10.42	4.27
Dehuiyou 50	3.43	1.18	26.76	97.29	43.58	9.30	13.24	0.89	31.26	3.00	11.24	5.44
Dehuiyou 903	4.32	1.79	26.61	86.52	39.52	8.62	15.63	0.76	42.93	3.63	5.91	2.99

Dexinyou 59	3.86	1.56	25.84	29.19	43.49	9.34	18.53	1.25	60.82	3.84	2.76	3.79
Deyou No.5	4.34	1.72	24.11	84.01	44.75	9.52	14.78	1.06	37.17	3.37	8.27	4.13
Deyou No.8	5.44	2.47	26.96	41.79	40.85	9.27	18.86	1.32	62.22	4.08	2.52	3.61
Deyou No.9	3.30	1.18	24.94	94.14	42.52	9.42	14.20	0.74	41.34	3.45	7.22	4.06
Deyouza 108	4.08	1.78	25.51	100.75	43.63	9.03	14.38	1.06	29.31	3.19	10.52	4.07
Fengyou 5103	3.17	1.18	25.60	41.12	43.05	9.80	18.84	1.32	60.77	4.02	4.00	3.76
Fengyou 701	4.96	2.15	30.45	103.98	35.79	10.53	21.29	0.95	58.26	4.44	2.61	4.83
Fengyou 730	2.84	1.05	22.16	36.78	44.84	11.01	21.17	1.40	57.72	4.18	3.19	4.00
Fengyou 737	2.16	1.88	23.88	34.49	44.42	9.25	18.90	1.42	63.19	4.10	3.68	3.76
Fengyou No.9	5.27	2.30	24.86	101.82	42.99	9.91	13.71	0.87	40.66	3.29	7.78	4.43
Fuyou 668	3.54	2.10	25.31	49.23	42.63	8.65	18.85	1.03	61.50	4.04	2.51	4.01
Ganyou 105	2.56	1.16	27.33	37.20	36.55	9.74	20.18	1.44	58.39	4.03	3.06	3.56
Ganyouza 108	4.65	2.31	25.23	57.32	41.92	10.66	18.18	1.19	48.61	4.08	5.97	4.31
Ganyouza No.2	3.35	1.70	26.95	29.48	40.82	7.44	19.85	1.26	64.26	4.08	3.48	3.58
Ganyouza No.5	2.92	1.68	27.47	43.46	37.18	8.92	19.45	1.19	62.84	4.11	3.41	5.15
Ganyouza No.6	4.45	1.27	25.42	101.05	44.83	9.92	14.18	0.77	29.54	3.26	9.24	3.45
Ganyouza No.7	4.19	2.23	27.01	37.05	40.09	9.27	20.17	1.27	61.10	4.00	2.35	4.54
Ganyouza No.8	3.71	1.90	23.42	43.87	46.61	10.61	13.72	0.91	69.25	3.39	1.33	6.36
Guoyou 1304	3.37	1.60	26.64	34.94	44.50	9.64	16.09	1.28	64.46	3.68	3.41	4.22
Heshengyou 555	4.06	2.05	25.52	42.08	41.00	10.20	19.20	1.06	63.34	4.07	1.29	4.27
Heshengyou 868	4.12	2.07	25.41	43.10	41.00	10.34	18.72	1.09	63.49	4.00	1.35	2.96
Heza 575	3.63	1.84	23.30	70.61	44.07	9.47	14.77	0.98	36.27	3.27	8.51	3.97
HN1	4.68	2.07	26.87	36.39	40.58	10.18	19.64	1.26	56.15	4.23	4.47	2.80
HN104	4.36	1.99	25.65	26.27	42.10	6.08	17.25	1.41	66.54	3.58	3.26	4.04
HN2	2.90	1.48	27.51	35.80	44.02	9.53	17.16	1.44	59.84	3.52	4.91	2.77

HN3	3.57	1.52	29.06	30.75	38.67	8.87	20.76	1.28	58.64	4.12	2.83	2.16
HN4	3.99	1.71	27.24	31.80	41.37	9.32	19.21	1.34	59.63	3.92	2.55	2.67
Huaganyou No.1	4.93	2.37	26.44	38.76	40.15	8.93	19.68	1.17	59.74	4.20	3.19	3.43
Huahang 901	3.77	1.75	18.79	38.48	51.29	11.33	16.57	1.21	62.28	3.83	1.97	3.83
Huashuang No.4	3.51	1.99	24.20	41.40	41.59	10.25	19.06	1.31	58.01	4.09	4.47	3.86
Huashuang No.5	4.83	2.02	24.36	49.57	41.92	10.32	17.48	1.29	59.95	4.10	5.72	4.08
Huawanyou No.4	4.59	1.85	25.21	93.72	44.31	9.47	15.72	0.99	29.51	3.29	9.96	4.14
Huayouza 62	3.60	1.67	27.41	30.04	40.28	10.68	20.19	1.31	59.22	4.04	2.30	4.24
Huayouza 95	2.72	1.07	22.55	37.51	44.76	11.94	23.52	1.13	55.36	4.48	1.27	4.21
Huayouza N0.10	4.04	1.60	27.27	39.02	38.86	10.26	19.29	1.15	60.88	4.24	2.36	4.27
Huayouza N0.12	4.03	2.57	23.78	44.70	39.80	10.47	21.01	1.20	56.81	4.49	1.77	3.96
Huayouza N0.13	3.59	1.77	25.85	39.40	38.81	8.65	21.05	0.95	64.49	4.47	0.96	3.78
Huayouza N0.14	3.43	1.63	25.46	39.46	39.49	9.00	20.45	0.86	63.22	4.40	0.57	3.95
Huayouza No.9	3.07	1.09	26.42	43.01	40.62	10.06	19.84	0.93	63.07	4.12	1.00	4.24
Huaza 291	2.79	1.79	25.57	32.56	40.66	10.18	21.68	1.56	61.93	4.05	2.00	4.70
Junlongyou No.5	3.93	1.81	23.53	107.01	44.79	10.46	14.03	0.84	32.73	3.40	9.17	4.38
Liangyou No.9	4.07	1.65	24.95	37.98	44.66	10.99	17.89	1.24	61.08	3.76	2.91	4.48
Mianxinyou 78	2.41	1.27	23.60	37.18	45.05	10.28	18.84	1.27	63.58	3.94	1.97	3.71
Mianyou No.11	3.92	1.49	25.02	54.67	44.80	8.23	15.55	0.71	24.78	3.27	11.32	3.89
Ningza No.19	4.46	1.91	26.36	31.13	41.32	8.73	19.61	1.39	63.41	4.15	3.49	3.24
Qingza No.7	2.67	1.38	25.27	35.62	44.61	11.92	20.11	1.32	58.42	3.97	2.53	3.51
Qinyou No.10	3.80	1.64	24.72	39.20	43.31	9.53	21.76	1.38	58.63	4.45	3.67	2.96
Qinyou No.7	4.14	1.38	31.37	39.07	34.87	10.37	23.31	1.21	54.67	4.73	3.03	4.52
Qinyou No.9	3.98	1.44	24.87	41.41	43.24	9.98	20.94	1.35	57.19	4.31	3.81	2.97
Rongyou No.18	5.16	1.63	25.48	26.44	44.72	9.24	19.05	1.35	61.41	4.05	3.08	3.69

RQ011	3.69	1.69	21.58	26.12	46.55	10.82	18.52	1.28	59.46	4.33	3.93	4.69
Shengguang 127	2.39	1.02	23.71	47.67	44.10	11.34	22.66	1.36	57.48	4.25	1.68	5.07
Shengguang 77	4.22	1.23	22.02	46.42	44.46	10.23	21.28	1.21	58.95	4.37	1.20	4.15
Shengguang 87	3.24	1.37	25.94	42.84	39.37	10.75	18.57	1.16	62.33	4.28	2.55	4.90
Shuangyou No.8	4.04	1.99	26.52	53.58	41.24	8.81	16.91	1.33	49.36	3.63	6.63	3.80
SWU45	3.44	1.78	18.40	31.67	49.09	11.41	19.64	1.35	60.86	4.57	1.85	3.02
T2159	3.01	1.36	20.68	70.69	45.93	9.54	15.28	1.07	35.62	3.33	8.46	3.82
Wenyou 99	3.62	1.93	26.30	42.47	44.76	10.12	17.97	1.40	60.14	3.93	4.59	4.28
Xiangyou No.15	4.67	2.71	24.07	35.05	46.09	10.14	18.05	1.21	64.33	3.84	1.95	4.31
Xiangyouza No.2	3.78	1.84	26.20	82.92	37.54	9.69	21.29	1.39	57.31	4.41	4.28	3.95
Xiwang 528F1	4.14	1.59	25.46	39.34	40.30	9.86	20.69	1.36	57.71	4.22	3.61	3.61
Xiwang 699	5.05	2.26	22.61	33.81	46.14	10.76	18.82	1.18	62.64	4.03	2.10	4.45
Yangguang 198	4.14	2.10	23.60	40.32	44.16	9.14	18.29	1.28	62.45	3.85	2.90	4.09
Yangguang 2009	3.79	2.06	23.75	39.71	44.47	9.17	18.06	1.26	62.28	3.83	3.14	4.07
YB3	3.64	1.71	20.99	51.00	48.37	11.24	21.32	1.33	55.90	4.50	4.09	5.18
Za 98033	4.48	1.96	26.12	38.32	40.92	7.85	19.26	1.38	64.48	4.11	2.70	3.69
Zheda 619	4.29	1.87	22.28	44.83	45.58	10.87	16.86	1.28	58.64	3.96	4.47	3.49
Zheshuang 758	4.14	1.89	23.19	86.69	43.86	9.29	16.20	1.20	39.59	3.60	8.33	3.57
Zheshuang No.3	4.31	2.19	23.22	55.00	44.57	9.78	18.69	1.30	58.09	4.05	3.96	3.83
Zheshuang No.8	4.71	2.14	19.29	26.32	47.65	6.95	13.67	0.65	70.00	3.94	3.26	3.50
Zheyou 50	4.33	2.19	24.70	37.26	49.86	11.56	20.31	1.23	56.38	4.24	3.82	4.91
Zhongduyou 998	3.49	1.71	24.21	105.98	43.42	10.55	13.62	0.85	38.08	3.28	7.48	4.41
Zhongnongyou 2008	3.51	1.78	27.24	32.51	37.88	9.03	19.97	0.92	65.96	4.46	0.66	4.14
Zhongshuang No.10	4.24	1.93	22.69	39.85	46.77	9.55	17.62	1.09	60.78	3.87	2.90	3.89
Zhongshuang No.11	5.03	2.16	23.38	36.42	47.07	9.80	15.18	0.81	66.75	3.62	0.81	4.29

Zhongshuang No.12	3.41	1.50	22.57	39.56	43.14	9.36	18.41	1.10	61.23	4.04	2.21	3.34
Zhongshuang No.5	2.94	1.27	23.15	51.24	41.86	8.97	19.38	1.25	57.45	4.11	3.11	3.07
Zhongshuang No.6	4.15	2.04	24.38	41.27	40.86	10.35	19.12	1.30	57.97	4.09	4.12	3.79
Zhongshuang No.7	3.71	1.78	20.15	36.33	46.02	10.69	20.29	1.26	58.75	4.29	3.89	4.71
Zhongshuang No.8	4.25	2.51	23.42	37.29	46.05	9.59	16.50	0.90	64.62	3.70	1.55	4.01
Zhongshuang No.9	3.84	1.72	24.91	34.33	42.52	9.57	20.86	0.94	60.89	4.35	1.39	3.88
Zhongyou 112	5.71	2.85	25.54	33.70	40.76	9.23	20.92	1.16	60.79	4.58	3.04	3.88
Zhongyou 36	4.50	2.57	24.13	37.17	48.83	9.72	17.34	1.21	62.39	3.69	1.67	3.45
Zhongyou 519	4.21	2.12	25.79	40.86	40.19	9.77	18.95	1.04	64.99	4.11	1.06	4.04
Zhongyou 589	5.77	2.51	23.38	42.85	45.43	9.66	16.96	1.12	62.75	3.80	2.47	4.56
Zhongyou 6766	3.89	2.10	23.43	41.04	42.32	10.00	17.29	1.21	60.69	3.68	2.59	3.85
Zhongyou 821	5.15	2.94	25.16	53.47	42.91	8.80	18.05	1.12	61.12	3.99	3.12	3.59
Zhongyouza 11	3.43	1.39	28.04	28.91	42.86	10.71	20.90	1.13	57.50	4.17	3.24	3.96
Zhongyouza 12	3.94	2.05	23.88	44.20	43.38	9.41	17.82	1.03	58.71	3.84	2.36	3.57
Zhongyouza 19	4.95	2.41	22.06	31.80	47.90	9.95	15.24	1.03	68.14	3.31	0.38	4.49
Zhongyouza No 1	3.11	1.43	28.14	37.61	39.60	10.90	20.95	1.23	57.69	4.38	3.96	4.02

Additional file 2: Primer sequence of the reference genes and candidate genes for qPT-PCR.

Gene ID	Forward Primer (5′→3′)	Reverse primer (5’→3’)
BnaC09g47620D	GACGGTGATGTTCTTCTT	CCTGCTTGTCCTTCATAG
BnaC08g12720D	GTGCTGCTATCTATCTGT	TACTTGTTCTTGTCTGTCT
BnaA05g34050D	AAGATATGTATGATGACGATGAA	ATAGCGAGTCTGTGAGTT
BnaC05g29840D	AGGATGAACAATAAGAAGA	AATCATTAGTCGTCTCTG
BnaC01g01860D	GCCGAGGAAGAAGAAGAC	GAGCAAGAAAGTCAATGATGTT
BnaC02g06710D	GGCTAAGAAGACTAAGGA	AGTAGGCATAATGAAGAGA
BnaC03g78230D	AGATGCTGCTGTTGTTAA	CTTAGACCTTGAGGAACTG
BnaC04g33060D	AGGTATTAGGTTGGAGGAAGA	AGTATCATCGTCACCATTCG
BnaA02g10660D	CATCTTGTGAGTGGTAGTAA	TAAGTGGATTGCCGTCTA
BnaA02g08130D	GGTCCTACAACTACTACGAAT	TCAGAGAAGCAACGAACT
BnaA06g03420D	TCGTCTGAGGAAGATTCT	GTCATCGCTACTGCTATC
BnaC03g29670D	GCTACGAACTACAAACAG	CCACGAACTCAGAGATAC
BnaA09g40900D	TTCCATACGCAGCAACAA	TCTCTTTCTTCACCATCTTACG
BnaA02g21230D	TCTGAGTTCGTGGTTATG	CGGCACATTCTTATCTTC
BnaA08g06270D	AGTAGTCGTCTTCAAGTA	TGTAATCCTAATCCTCGTA
BnaA08g25110D	ATGCTTCGTGTTGGTGAT	AATCCTTCTCAAGAGTCTCATC
BnaA01g14400D	GATAGTGATGGCGTTGGA	CTCTTGAATCGTCGTAATGTT
BnaC07g26240D	GGCAAGGAGAAGAAGGTTA	TTGTTGTCGCAGAGGTAG
BnaA03g08800D	AGGCTAAGAAGACTAAGG	GTTGGCATAATGAAGAGA
BnaC08g45610D	TGTTCCGTTATCTCTTATTCC	CGCCAGGTAGTATAGTCT
BnaA04g15290D	CCTCAGTATGGTCAGTCA	CGTAATAATCATAATCACCTTGT
BnaA03g00380D	GGTTCAGGTATCTTCAAG	GTCACTGTAATGAGTCAC

Additional file 3: Throughput and quality of RNA-seq for the 18 libraries ofGanyouza No.5. The LT and CK indicate germination treatment under the low temperature and normal temperature, respectively.

Sample	Raw data		Clean data		Q30 (%)	Ratio of valid reads (%)
	Base (bp)	Reads Number	Base(bp)	Reads Number		
LT-1d	4414827900	29432186	4349664717	29085360	91.84	98.82
LT-1d	4488426000	29922840	4416990859	29547158	91.72	98.74
LT-1d	3922709100	26151394	3859398517	25812898	91.6	98.71
CK-1d	4370804400	29138696	4299096482	28748920	91.52	98.66
CK-1d	5321328900	35475526	5222365607	34923964	90.88	98.45
CK-1d	5148978600	34326524	5065846372	33905606	91.92	98.77
LT-2d	4102443300	27349622	4039238339	27015970	91.84	98.78
LT-2d	4932360900	32882406	4860402464	32498240	91.99	98.83
LT-2d	4099739700	27331598	4039794286	27011000	92.06	98.83
CK-2d	4593468600	30623124	4522264286	30241252	91.65	98.75
CK-2d	4841014500	32273430	4770445268	31902078	91.98	98.85
CK-2d	4099084500	27327230	4037890951	27005034	91.87	98.82
LT-3d	4242447300	28282982	4175085813	27925034	91.76	98.73
LT-3d	5094772200	33965148	5024279432	33604186	92.31	98.94
LT-3d	4611284700	30741898	4538119008	30355482	91.78	98.74
CK-3d	4397909100	29319394	4319284319	28890280	91.07	98.54
CK-3d	4599842100	30665614	4530884919	30307820	91.9	98.83
CK-3d	4155921900	27706146	4085013846	27323422	91.43	98.62

Additional file 4: Throughput and quality of RNA-seq for the 18 libraries of Huawanyou No.4. The LT and CK indicate germination treatment under the low temperature and normal temperature, respectively.

Sample	Raw data		Clean data		Q30	Ratio of valid
	Base(bp)	Reads Number	Base (bp)	Reads Number	(%)	reads (%)
LT-1d	4187365800	27915772	4150452676	27738296	93.86	99.36
LT-1d	4151149500	27674330	4105575151	27488382	93.93	99.33
LT-1d	3899643000	25997620	3862050281	25824266	93.89	99.33
CK-1d	3595458300	23969722	3555602928	23765456	93.69	99.15
CK-1d	4002381900	26682546	3958968936	26477222	93.85	99.23
CK-1d	3669741600	24464944	3626497723	24241230	93.52	99.09
LT-3d	3960329400	26402196	3918875407	26190742	93.52	99.2
LT-3d	3894786900	25965246	3854811666	25760102	93.9	99.21
LT-3d	4363171200	29087808	4320531561	28876002	94.04	99.27
CK-3d	3720571500	24803810	3686452323	24633976	93.9	99.32
CK-3d	4634362500	30895750	4594820740	30714214	94.24	99.41
CK-3d	4048966800	26993112	4010177981	26804162	93.98	99.3
LT-5d	4403937000	29359580	4367731221	29189312	94.32	99.42
LT-5d	4200576900	28003846	4157167802	27779534	93.81	99.2
LT-5d	4085359200	27235728	4042786989	27021904	93.62	99.21
CK-5d	4075603200	27170688	4040436397	26992274	93.89	99.34
CK-5d	4195327800	27968852	4159006723	27798442	94.18	99.39
CK-5d	3432098700	22880658	3395012818	22703822	92.84	99.23

Additional file 5: Gene dendrogram obtained by average linkage hierarchical clustering. The gene dendrogram and module assignment are shown by the hierarchical clustering diagram. The color rows underneath the dendrogram shows the module assignment determined by the Dynamic Tree Cut and Merged Dynamic. The program algorithm firstly pre-clusters the different expressed genes into large clusters by using a variant of k-means clustering as shown in the top colorful row. The cluster-merging step is performed to merge modules whose eigengenes are high correlated, as shown in the bottom colorful row.

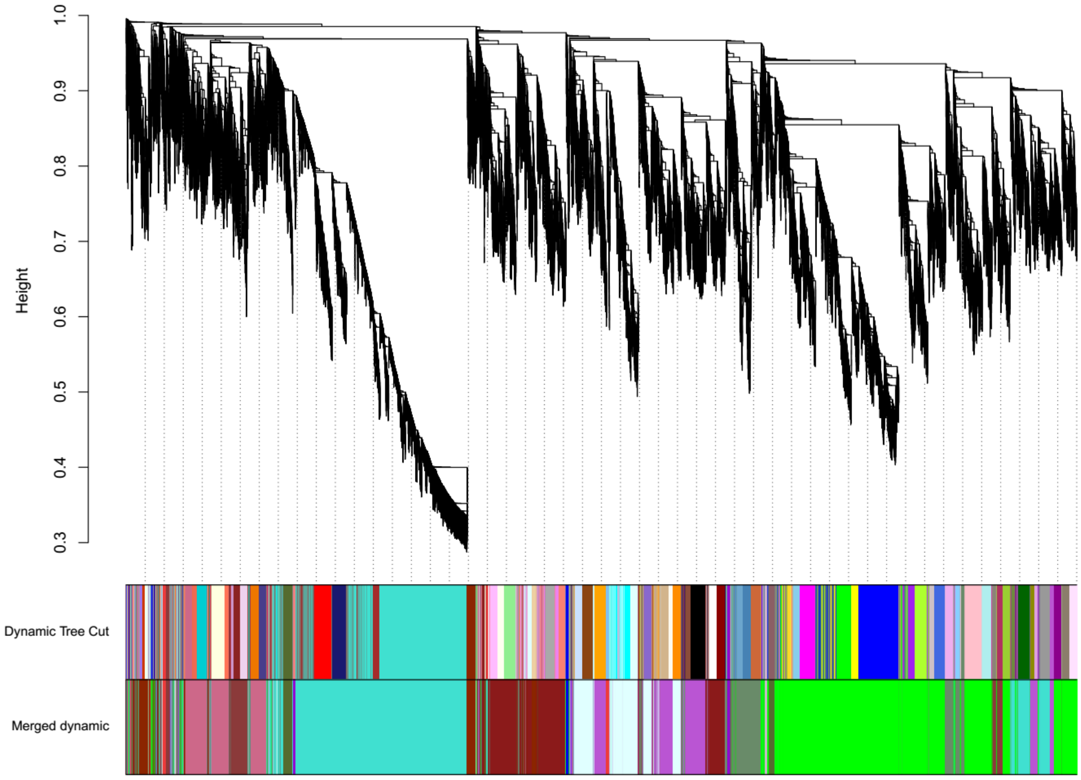

Additional file 6: Topological overlap matrix for the co-expression network. Topological overlap measure, a pairwise measure of node similarity indicates how close the neighbors of a gene are to the neighbors of the other gene. In the heatmap, each row and column corresponds to a gene, light color denotes low topological overlap, and progressively darker red denotes higher topological overlap. Darker squares along the diagonal correspond to modules. The gene dendrogram and module assignment are shown along the left and top.

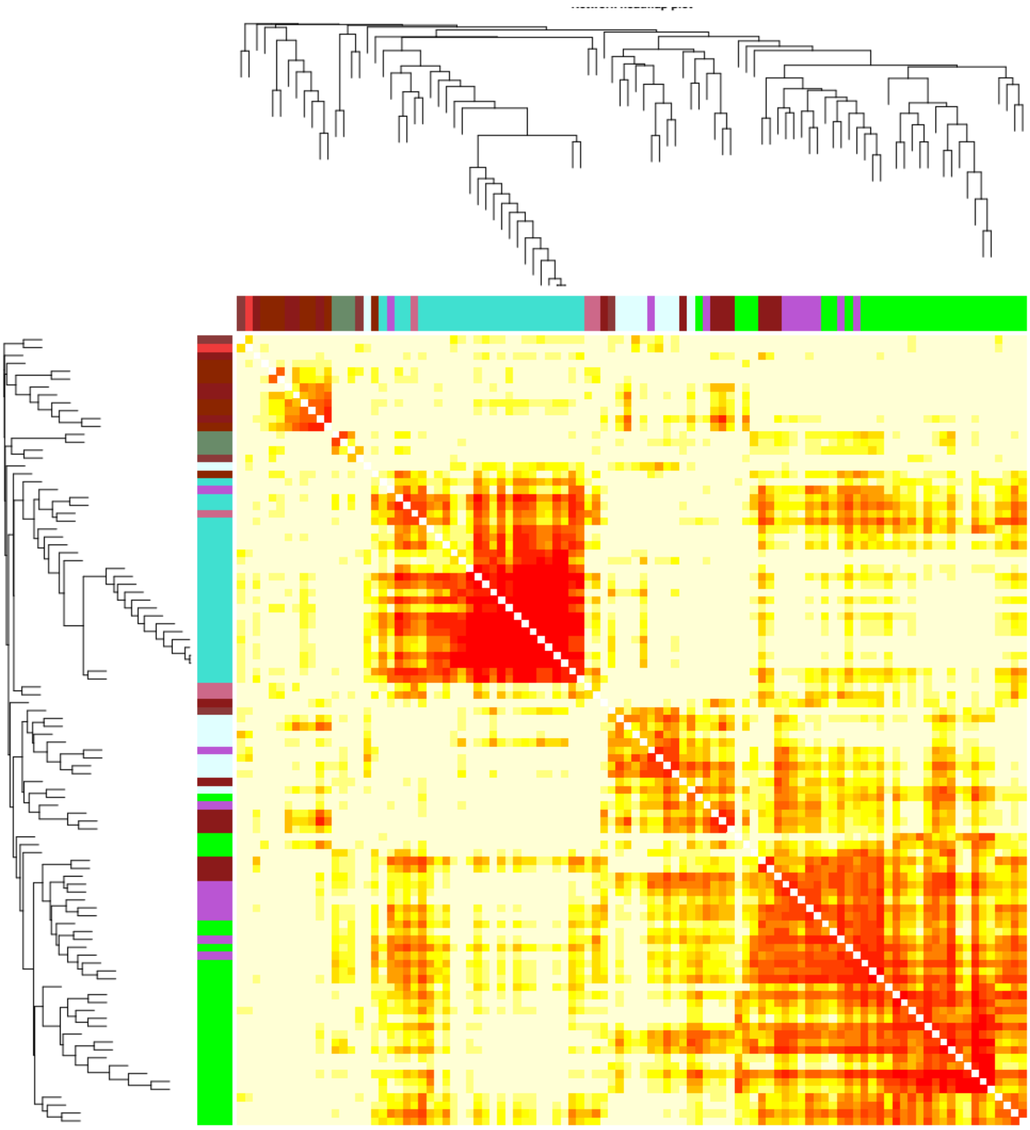

Additional file 7: The venn diagram of up-regulated genes (A) and down-regulated genes (B) for FAG. The FAG represents the genotype Ganyouza No.5, which have a fast germination speed under low temperature condition.

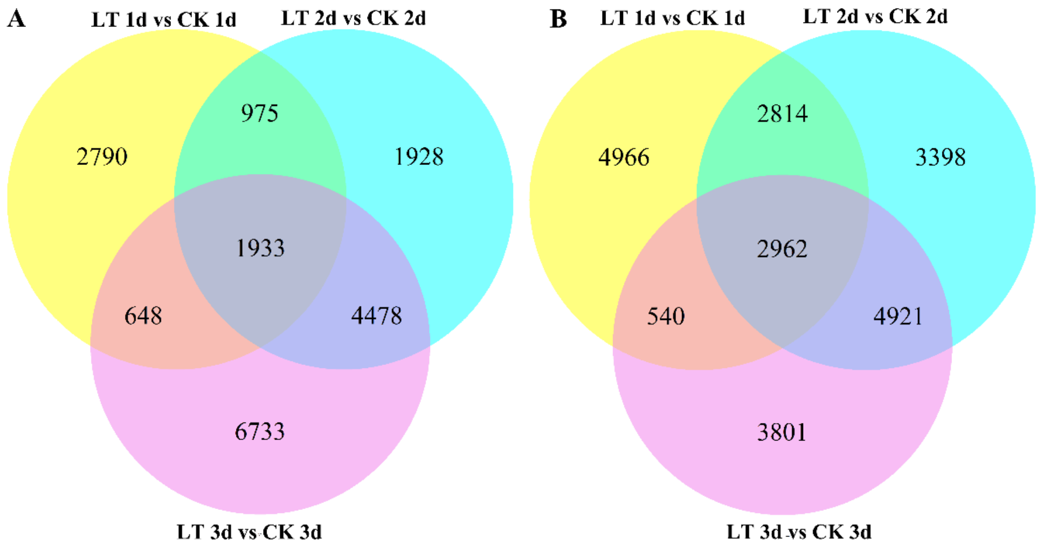

Additional file 8: The venn diagram of up-regulated genes (A) and down-regulated genes (B) for SLG. The SLG represents the genotype Huawanyou No.4, which have a slow germination speed under low temperature condition.

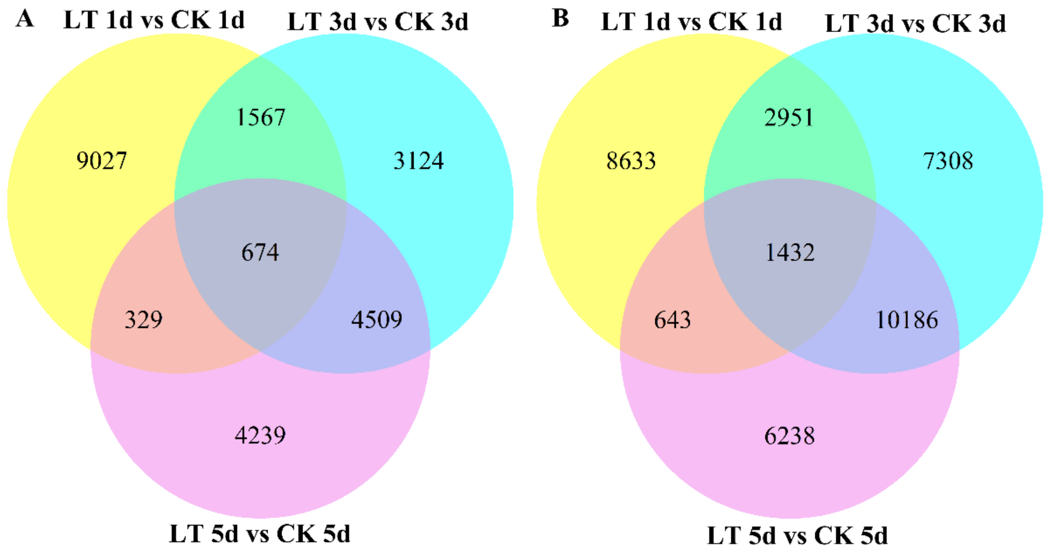

Supplement: Supplementary file 1 — Additional file [file 41598_2018_37099_MOESM1_ESM.pdf]
